# Evaluating the efficacy and safety of GKT137831 in adults with type 1 diabetes and persistently elevated urinary albumin excretion: a statistical analysis plan

**DOI:** 10.1186/s13063-020-04404-0

**Published:** 2020-06-03

**Authors:** Alysha M. De Livera, Anne Reutens, Mark Cooper, Merlin Thomas, Karin Jandeleit-Dahm, Jonathan E. Shaw, Agus Salim

**Affiliations:** 1 Baker Heart and Diabetes Institute, Melbourne, VIC 3004 Australia; 2 The University of Melbourne, Parkville, VIC 3010 Australia; 3 Monash University, Clayton, VIC 3168 Australia; 4 Latrobe University, Bundoora, VIC 3086 Australia

**Keywords:** Statistical analysis plan, Randomised controlled trial, GKT137831, Albuminuria, Type 1 diabetes

## Abstract

**Background:**

The investigational medicinal product GKT137831 is a selective inhibitor of NOX 1 and 4 isoforms of the nicotinamide adenine dinucleotide phosphate (NADPH) oxidase family of enzymes, which has the potential to ameliorate diabetic kidney disease. An investigator-initiated, double-blind, randomised, placebo-controlled, multicentre phase 2 clinical trial started recruitment in December 2017, with the aim of evaluating the efficacy and safety of GKT13783, in adults with type 1 diabetes mellitus and persistently elevated urinary albumin excretion over a period of 48 weeks.

**Methods/design:**

The trial is currently recruiting in Australia and New Zealand, with recruitment expected to end on 30 June 2020. The primary outcome measure of the trial is the urinary albumin excretion level measured at 48 weeks of treatment. This statistical analysis plan presents an update to the published trial protocol and provides a comprehensive description of the statistical methods that will be used for the analysis of the data from this trial. In doing so, we follow the “Guidelines for the content of statistical analysis plans in clinical trials” to support transparency and reproducibility of the trial findings.

**Discussion:**

With the use of this prior statistical analysis plan, we aim to minimise bias in the reporting of the findings of this trial, which evaluates the investigational medicinal product GKT137831. The results of the trial are expected to be published in 2022.

**Trial registration:**

ANZCTR registry: ACTRN12617001187336. Registered on 14 July 2017.

Universal Trial Number: U1111-1187-2609; Protocol number: T1DGKT137831; Genkyotex trial number: GSN000241.

## Introduction

The kidney is a major target organ of microvascular damage in type 1 diabetes mellitus. Diabetic kidney disease affects 14–31% of people with type 1 diabetes mellitus [1], with studies showing that there is an urgent need for innovative treatment strategies to prevent, arrest, treat and reverse diabetic kidney disease [2].

The active investigational medicinal product evaluated in this study, a NOX1/4 inhibitor known as GKT137831 or setanaxib, has the potential to ameliorate diabetic kidney disease, and has been previously evaluated in people with type 2 diabetes mellitus and overt nephropathy (NCT02010242). The present trial is a separate study in people with albuminuria and type 1 diabetes mellitus [2] with a longer treatment duration and a higher dose. The overall objective of this trial is to evaluate the efficacy and safety of GKT137831 400 mg, taken twice a day, compared to placebo in adults with type 1 diabetes mellitus and elevated urinary albumin excretion.

Here, we provide a detailed description of the statistical methods that will be used for analysing the data obtained from this trial. In doing so, we followed the Guidelines for the content of statistical analysis plans in clinical trials [3], and care was taken to include the necessary information and sections as per these guidelines. This statistical analysis plan uses the recently published study protocol [2]. With the use of this statistical analysis plan, we aim to minimise reporting bias, supporting both transparency and reproducibility of the trial findings.

## Study methods

### Trial design

**Fig. 1:**
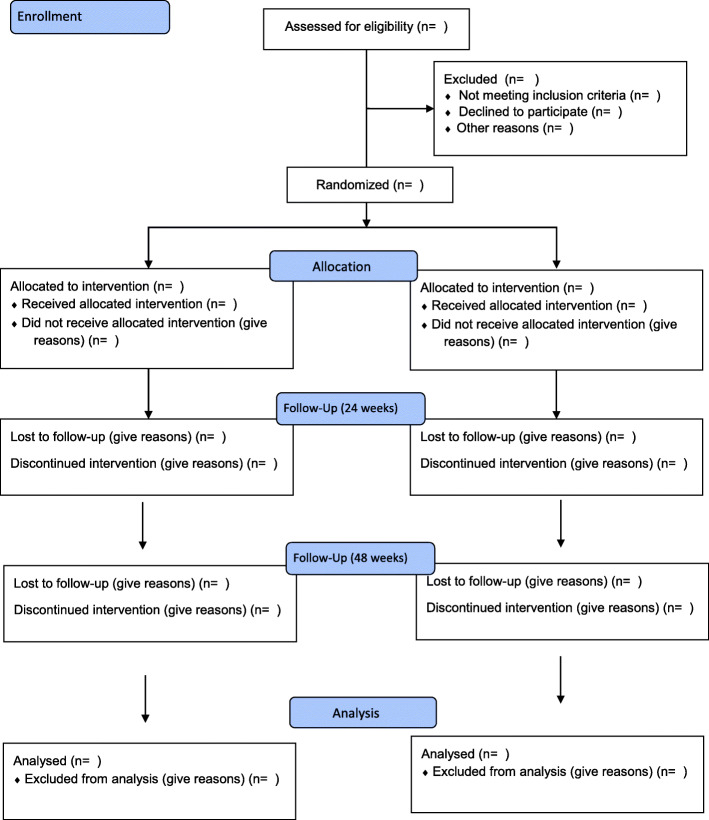
A flow diagram of the trial based on Consolidated standards of reporting trials (CONSORT) 2010

This is a multicentre, multi-national, phase 2, randomised, double-blind, placebo-controlled clinical trial with two parallel arms with 1:1 allocation ratio, which will test the effect of oral GKT137831 400 mg taken twice a day compared to placebo, on urine albumin to creatinine ratio, in adults with type 1 diabetes mellitus and persistent albuminuria despite optimal preceding standard of care treatment. The trial started recruitment in December 2017 and the recruitment is expected to end on 30 June 2020. The treatment period is 48 weeks with total study duration for the participant of up to 56 weeks. Figure 1 and Table 4 of the protocol [2] describe, respectively, the timeline for the study, which consists of 10 study visits in total, and the schedule of events.

### Randomisation

The randomisation ratio is 1:1 between the active drug (GKT137831) and placebo control arms, and is stratified by study centre and urinary albumin–creatinine ratio (UACR) level with the grouping defined as ≤ 35 mg/mmol or > 35 mg/mmol in female participants and ≤ 25 mg/mmol or > 25 mg/mmol in male participants using a minimisation strategy [4]. Participants will be randomised using a minimisation program integrated into the Interactive Web Response System, and is accessed live by sites through the website Randomize.net (see Sect. 3.3 of the protocol [2]).

### Sample size

Up to 284 participants in total are expected to be screened (anticipated screen fail rate up to 50%) with 142 participants each randomised to the treatment and placebo groups, to achieve 120 participants reaching the end of the treatment period in total (60 per treatment group). Sect. 2.5 of the protocol [2] provides details of the sample size calculations. In brief, the assumptions of the sample size calculations were the following: mean UACR of 13.56 mg/mmol in the control arm, difference between UACR means in the treatment arm of 3.56 mg/mmol/L (26% reduction in mean UACR in the treatment arm), equal standard deviations of 7.5 mg/mmol in the two arms, and within-subject correlation of 0.3. Using mixed model analysis of covariance with log-transformed UACR as the outcome, several scenarios were examined: 60 participants per treatment arm are required to achieve power > 90% with an alpha level of 0.05 and two-sided hypothesis testing, and 40 participants per treatment arm are required to achieve power > 90% with an alpha level of 0.05 and one-sided hypothesis testing.

### Trial framework

The primary analysis at the end of this trial will be a superiority comparison of GKT137831 versus placebo, where the null hypothesis to be tested for the primary and secondary endpoint analyses is that the effect of GKT137831 is no different to placebo and the alternative hypothesis is that GKT137831 is superior to placebo. The statistical hypotheses are described in detail in “Hypotheses” section. For the primary outcome, the minimal clinically important difference will be considered according to the sample size calculations in “Sample size” section; that is as a difference between UACR means in the treatment arms of 3.56 mg/mmol/L.

### Statistical interim analysis

There are no planned interim analyses.

### Timing of final analysis

All outcomes will be analysed collectively at the end of the trial at 48 weeks.

### Timing of outcome measures

The expected visit dates and visit windows are presented in Figure 1 of the protocol [2], and Table 4 of the protocol [2] describes the timing of all outcome measures.

## Statistical principles

### Confidence intervals and significance level

As the trial investigators have prior clinical knowledge about the directions of the effects on the endpoint UACR (primary outcome), midpoint UACR, endpoint and midpoint estimated glomerular filtration rate (eGFR) (secondary outcomes), the statistical tests for the primary and secondary hypotheses will be one-sided. For these outcomes, based on all the literature to date, there is no evidence to suggest that the placebo group will have a better outcome than the intervention group. The statistical tests for the safety and exploratory analyses will be two-sided. All the statistical tests will be performed using a 5% significance level, and we will report the 95% confidence interval. No adjustment for multiplicity is needed for the primary hypothesis. Testing for multiple comparisons in the secondary and exploratory hypotheses will be accounted for by adjusting the *p* values obtained from these analyses using the Benjamini-Hochberg method [5].

### Adherence and protocol deviations

#### Definition of adherence

Study drug treatment adherence for each visit interval is defined as taking at least 80% of the required study drug dosage. For each participant, this will be based on the percentage compliance calculated as: percent compliance = (number of capsules taken by the participant/number of capsules the participant should have taken) *100%. In this study, the participants will be taking four capsules twice a day.

#### Presentation of adherence

The number and percentage of participants taking more than 80% of the required study drug will be presented by treatment arm and visit (see Table 1), and the descriptive statistics on the percentage compliance will also be presented. No formal statistical testing will be performed.

**Table 1:** The number and percentage of participants taking more than 80% of the prescribed treatment

Visit number^a^	Treatment group	
	GKT137831 N (%)	Placebo, *N* (%)
4		
5		
6		
7		
8		
9		
10		

^a^ Visit number refers to Figure 1 of the protocol [1]

#### Definition of protocol deviations

Protocol deviations, “any change, divergence, or departure from the study design or procedures in the protocol [2]”, are defined according to the International Conference on Harmonisation of Technical Requirements for Registration of Pharmaceuticals for Human Use (ICH) guideline E3 on Structure and Content of Clinical Study Reports July1996 [6], classified as either “important” or “non-important”. Important protocol deviations are deviations that may significantly impact the completeness, accuracy, and/or reliability of the study data or that may significantly affect a subject’s rights, safety, or well-being. Non-adherence as defined in “Definition of adherence” section will be considered an important protocol deviation. Deviations that do not fit this definition are non-important.

#### Presentation of protocol deviations

Protocol deviations will be summarised by treatment arm, using the number and percentage of participants with important and non-important protocol deviations, and details on the type of deviation. The percentages will be calculated based on the participants in the intention-to-treat (ITT) analysis set as the denominator. No formal statistical testing will be performed. The details of protocol deviations including adherence will be added as an aide memoire to Fig. 1.

### Analysis populations

The following four analysis sets will be used in the study.

#### Main analysis set (ITT analysis set)

The ITT analysis set is the main analysis set that will be used for evaluating the primary hypothesis, and it consists of all randomised participants with each participant retaining their treatment group as originally allocated by the randomisation procedure.

#### Modified intention-to-treat analysis set

The modified ITT analysis set consists of participants who have been randomised and have taken at least one capsule of investigational product or placebo, with each participant in the set retaining their treatment group as originally allocated by the randomisation procedure.

### Safety analysis set

The safety analysis set consists of all participants who have taken at least one capsule of investigational product or placebo and have at least one subsequent safety assessment, which is described in Sect. 4.2 of the protocol [2].

#### Per protocol (PP) analysis set

The PP analysis set consists of the subset of participants who completed the 48-week treatment period and do not have an important protocol deviation.

## Trial population

### Screen data

At enrolment, the following summaries will be presented by study centre and overall for all screened participants: number of participants screened, the number of recruited participants, the number of screened and eligible participants who are not recruited, the reason for non-recruitment, and the number of recruiting days.

### Eligibility criteria

Participant inclusion and exclusion criteria are presented in Tables 2 and 3 of the protocol [2], respectively. The participants with type 1 diabetes mellitus need to have persistent albuminuria, documented abnormal UACRs in the preceding 24 months before enrolment in the trial, already be receiving standard of care therapy for albuminuria, and have well-controlled blood pressure and HbA1c. Participants are excluded if there is occurrence within 13 weeks before screening of recent changes in medications that may alter albumin excretion or eGFR, or certain procedures that may potentially affect kidney function, or acute kidney injury.

### Recruitment

The trial profile will be illustrated using a flow diagram based on the Consolidated Standards of Reporting Trials) 2010 (CONSORT) [7] as shown in Fig. 1. The following information will be provided in the diagram summarizing the number of participants who are eligible at screening, not eligible at screening (with reasons provided), eligible and randomised, eligible but not randomised (with reasons provided), received the randomised allocation, did not receive the randomised allocation (with reasons provided), withdrawals/lost to follow-up (with reasons provided - see “Withdrawals/follow-up” section), randomized and included in the primary analysis, and randomised and excluded from the primary analysis (with reasons provided).

### Withdrawals/follow up

The number of participants at each level of withdrawal will be documented and the timing of withdrawals will be presented in the CONSORT diagram in Fig. 1, categorized as (1) withdrawn from the intervention but continued with follow up, (2) withdrawn from follow up but allowed data collected to date to be used, (3) withdrawn from follow up and withdrawn consent for data collected to date to be used, and (4) lost to contact/follow up.

### Baseline participant characteristics

The characteristics of the participants, will be summarised by study arm and overall at baseline using summary statistics as shown in Table 2. Continuous variables will be summarised by the appropriate central tendency and dispersion measures, using either mean and standard deviation (SD) or median and 25th–75th percentile. Categorical data will be summarised using frequencies and percentages. The frequencies and percentages of missing values will be reported for all variables.

**Table 2:** The baseline characteristics of the participants by intervention, placebo groups and overall

Baseline characteristic	Intervention (GKT137831) group (*n* = xxx)		Placebo group (*n* = xxx)		Percentage standardised difference	Overall (*n* = xxx)	
	Values	Missing, *n* (%)	Values	Missing, *n* (%)		Values	Missing, *n* (%)
**Urine albumin–creatinine ratio (UACR)** (log-transformed)							
Mean (standard deviation (SD))							
**Estimated glomerular filtration rate (eGFR)** (log-transformed)							
Mean (SD)							
**Gender**							
Male, *n* (%)							
Female, *n* (%)							
**Age** (years),							
Mean (SD)							
**Ethnicity**							
xx, *n* (%)							
Other, *n* (%)							
**Clinical variables**							
Systolic blood pressure (mmHg), mean (SD)							
Diastolic blood pressure (mmHg), mean (SD)							
Use of angiotensin-converting-enzyme inhibitor (ACEi), *n* (%)							
Use of angiotensin II receptor blocker (ARB), *n* (%)							
Body mass index (kg/m^2^), mean (SD)							
Duration of diabetes mellitus at baseline (years), mean (SD)							
Glycated haemoglobin (HbA1c %), median (25th–75th percentile)							

## Analysis

### Outcome definitions

The outcome measures of the GKT137831 trial can be divided into primary, secondary, exploratory, pharmacokinetics, and safety outcome measures. This document focuses on the primary, secondary, exploratory, and safety outcomes. Pharmacokinetics assessments will be reported elsewhere.

#### Primary outcome measure

The primary outcome measure of the trial is the urinary albumin excretion level measured by the urine albumin–creatinine ratio (UACR) in milligrams per millimole at the end of the treatment (endpoint UACR), and is defined as the geometric mean of four UACR values, which consists of the two consecutive, daily, first void UACR values at 46 weeks of treatment and the two consecutive, daily, first void UACR values at 48 weeks of treatment. Baseline UACR is defined as the geometric mean of four UACR values, which consists of the two consecutive, daily, first void UACR values at 2 weeks before randomisation and the two consecutive, daily, first void UACR values at randomisation. In the statistical analyses, endpoint UACR will be adjusted for baseline UACR (see “Primary hypothesis” section).

#### Secondary outcome measures

The secondary outcome measures of the trial are the following:

##### Midpoint urine albumin–creatinine ratio

The urinary albumin excretion level at the midpoint of the treatment period measured by the urine albumin–creatinine ratio (midpoint UACR), defined as the geometric mean of the two consecutive, daily, first void UACR values taken at the midpoint of treatment at 24 weeks of treatment.

##### Endpoint estimated glomerular filtration rate

The eGFR at the end of the treatment period at 48 weeks, measured in millilitres per minute relative to body surface area (endpoint eGFR), and is defined as the geometric mean of eGFR at week 46 of treatment and the eGFR at week 48 of treatment. Baseline eGFR is defined as the geometric mean of the eGFR at 2 weeks before randomisation and the eGFR at randomisation.

##### Midpoint estimated glomerular filtration rate

The eGFR at the midpoint of the treatment period at 24 weeks, measured in millilitres per minute relative to body surface area (midpoint eGFR), and is defined as the log-transformed eGFR values obtained at week 24 of treatment.

#### Exploratory outcome measures

The exploratory outcome measures obtained at the end (at 48 weeks) of the treatment period are presented in Sect. 4.2.5 of the protocol [2] and are summarised in Supplementary Table 1. In the statistical analyses, the endpoint exploratory outcome measures will be adjusted for their baseline values taken at randomisation (see “Exploratory analyses” section).

#### Safety outcome measures

The key safety outcome measures are treatment-emergent adverse events and abnormal laboratory analytes as defined in the protocol [1] Sect. 10.4.4.2 and 10.4.4.3, respectively. All safety outcome measures are given in Supplementary Table 2 along with references to the sections where they are defined in the protocol [1].

### Hypotheses

#### Primary hypothesis

The null hypothesis is that the population mean of the endpoint UACR in the intervention group is the same as that in the placebo group (after adjustment for baseline UACR). The alternative hypothesis is that the population mean of the endpoint UACR is lower in the intervention group, compared to that in the placebo group (after adjustment for baseline UACR*)*.

#### Secondary hypotheses

##### Midpoint urine albumin–creatinine ratio

The null hypothesis is that the population mean of the midpoint UACR in the intervention group is the same as that in the placebo group (after adjustment for baseline UACR). The alternative hypothesis is that the population mean of the midpoint UACR is lower in the intervention group, compared to that in the placebo group (after adjustment for baseline UACR).

##### Endpoint estimated glomerular filtration rate

The null hypothesis is that the population mean of the endpoint eGFR in the intervention group is the same as that in the placebo group (after adjustment for baseline eGFR). The alternative hypothesis is that the population mean of the endpoint eGFR is higher in the intervention group, compared to that in the placebo group (after adjustment for baseline eGFR).

##### Midpoint estimated glomerular filtration rate

The null hypothesis is that the population mean of the midpoint eGFR in the intervention group is the same as that in the placebo group (after adjustment for baseline eGFR). The alternative hypothesis is that the population mean of the midpoint eGFR is higher in the intervention group, compared to that in the placebo group (after adjustment for baseline eGFR).

#### Exploratory concepts

The exploratory outcomes (summarised in Supplementary Table 1) taken at the end of the treatment period will be compared between the treatment and placebo groups (adjusting for the values taken at the beginning of the treatment). These will be carried out as exploratory analyses to inform future research directions.

#### Safety objectives

The safety objectives are to assess the effects of the GKT137831 compared to placebo on the key safety outcome measures (treatment-emergent adverse events and abnormal laboratory analytes) and the other safety outcome measures defined in “Safety outcome measures” section.

### Analysis methods

#### Primary analyses

##### Main analysis model

The main analysis will be based on the ITT analysis set. The analysis model will be a linear mixed effects model with endpoint UACR (log-transformed) as the outcome, a categorical variable indicating the allocated grouping structure (intervention versus placebo) as the exposure, and a random effect term to account for the study centre variability. The model will incorporate other covariates as discussed in “Covariate adjustment” section. The results from this analysis will be summarised in Table 3.

**Table 3:** Primary and secondary results of the GKT137831 trial

Outcome		Analysis	Mean (standard deviation)		Estimated mean difference^a^	95% Confidence Interval	*P* value
			Intervention (GKT137831) group (n = xxx)	Placebo group (*n* = xxx)			
Primary	Endpoint UACR	Intention to treat (ITT)					
		modified ITT					
		Per protocol (PP)					
Secondary	Midpoint UACR	Intention to treat (ITT)					
		modified ITT					
		Per protocol (PP)					
	Endpoint eGFR	Intention to treat (ITT)					
		modified ITT					
		Per protocol (PP)					
	Midpoint eGFR	Intention to treat (ITT)					
		modified ITT					
		Per protocol (PP)					

*UACR*  urine albumin: creatinine ratio, *eGFR* estimated glomerular filtration rate

^a^ In the intervention group compared to placebo

##### Covariate adjustment

The main analysis model described in “Main analysis model” section will include (log-transformed) baseline UACR as a covariate. To assess potential baseline covariate imbalance, the participant characteristics at baseline described in “Baseline participant characteristics” section will be compared between the intervention and placebo arms. Instead of using hypothesis testing for this purpose due to its dependence on sample size and emphasis on statistical significance, the standardized differences in means or proportions [8] will be used. The main analysis model will also include the baseline variables that have values of 10% or greater, in the magnitude of the percentage standardised differences, as covariates*.*

##### Checking assumptions in statistical models

The assumptions of linearity between the explanatory variables and the outcome, constant variance and independence of the errors, and normality of the error distribution will be checked using plots of observed values versus predicted values, residuals versus predicted values, residuals versus individual independent variables, and normal probability plots. These plots will also be used to detect any outliers.

##### Alternative methods to be used if distributional assumptions do not hold

The UACR levels used in the analysis model will be log-transformed. However, alternative transformations will be considered in the cases where the assumptions of constant variance or normality of the error distribution is violated. Non-linear terms will be incorporated to account for any non-linearity between the explanatory variables and the outcome. If it is verifiable that any of the outliers are due to data that are incorrectly measured or registered, these outliers will be removed. The remaining outliers will be accommodated using robust estimation of linear mixed effects models [9].

##### Subgroup analyses

Potential effects of the following pre-specified subgroups on the endpoint UACR levels will be tested using the model described for the main analysis in “Main analysis model” section extended to include the subgroup and subgroup by treatment interactions: (1) gender, (2) baseline age group (younger than the median and equal to or older than the median), (3) duration of diabetes mellitus at baseline (shorter than the median and equal to or longer than the median duration), (4) body mass index (BMI) at baseline (lower than the median and equal to or higher than the median value), and (5) glycated haemoglobin (HbA1c) level at baseline (lower than the median and equal to or higher than the median value). Likelihood ratio tests will be used to test for interactions and the results will be presented on forest plots with the interactions results alongside. The results will also be presented as outlined in Table 4.

**Table 4:** Subgroup analyses in the intention-to-treat group, to be presented as forest plots

Subgroup	Number (%)		Estimated mean difference^a^	95% Confidence Interval	*P* value
	Intervention (GKT137831) group (*n* = xxx)	Placebo group (*n* = xxx)			
Sex					
Male					
Female					
Age					
< median					
≥ median					
Diabetes mellitus duration					
< median					
≥ median					
BMI					
< median					
≥ median					
HbA1c					
< median					
≥ median					

*BMI* body mass index, *HbA1c* glycated haemoglobin

^a^ In the intervention group compared to placebo

##### Missing data methods

An investigation of the patterns of missing data will first be carried out. A summary of which variables contain missing values, proportion of missing values in each variable, and patterns and predictors of missingness will be provided. This summary will then be used to decide on the most appropriate method to handle missing data. If the missing values only arise in the dependent variable, no imputation will be carried out, as the main analysis model, which is a linear mixed effects model, is robust against the missing-at-random (MAR) mechanism. However, if the missing values are present in the covariates, following the trial protocol, if suitable [10], missing values will be handled using multiple imputation [11] considering both bias and precision [12]. The following steps will be undertaken: the missing data in the analysis set will first be assessed by calculating the proportion of missing values, investigating the patterns of missingness in the variables, and identifying predictors of missingness. Based on this, a suitable imputation model that typically consists of all the variables in the analysis model and additional auxiliary variables (i.e., the variables that correlate with the variable with missing values and/or are predictors of missingness) [13] will be used to generate 50 completed datasets with imputed values using a fully conditional specification [14]. If missing values are present in the composite measures (e.g., endpoint UACR and baseline UACR, which are overall measures based on multiple log-transformed UACRs) due to item-level missingness, multiple imputation will be carried out at the item level [15]. The analysis model will then be used in each completed dataset and the results will be combined using Rubin’s rules [16].

##### Sensitivity analyses

**Fig. 2:**
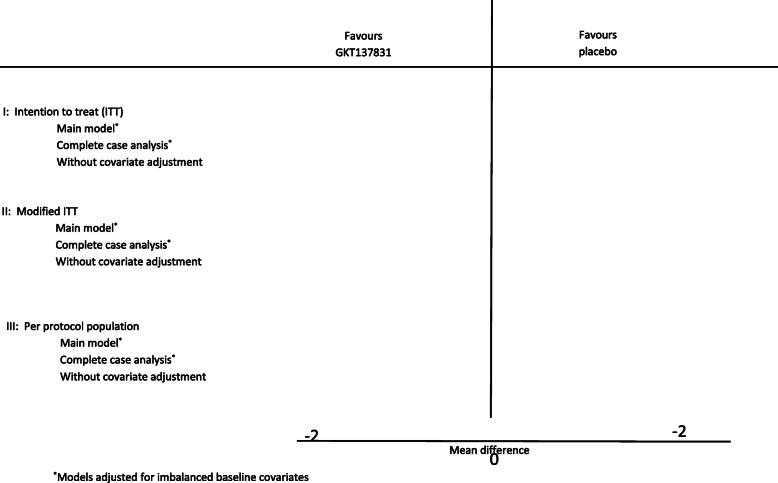
Forest plot of the mean differences (95% confidence intervals) in the endpoint urine albumin–creatine ratio (UACR) (intervention versus placebo group)

The results obtained by using a mixed effects model of the (log-transformed) endpoint UACR with the allocated grouping structure as the exposure, a random effect term to account for the study centre variability, only incorporating (log-transformed) baseline UACR as a covariate without additional baseline covariates will also be made available. In addition, the sensitivity of the results to the decisions made in constructing the imputation model, will be investigated using visualisations of the distributions of imputed and observed values, standard regression diagnostics [17], and comparisons with complete case analysis. The results will be presented as outlined in Fig. 2.

##### Additional analyses

Supplementary analyses will be completed by repeating the main analysis described in “Main analysis model” section - “Sensitivity analyses” section using (1) the modified ITT and (2) the PP analysis sets, and these results will be reported in the proposed format.

#### Secondary analyses

The analyses described in “Main analysis model” section - “Additional analyses” section will be repeated:

1. For the secondary outcome measures defined in “Secondary outcome measures” section, and
2. Using the subsets of the GKT137831 group who have taken the 800 mg/day drug for (a) > 6 months and (b) > 12 months as the intervention group for all primary and secondary outcomes defined in “Primary outcome measure” section and “Secondary outcome measures” section, respectively.

The results will be reported in the proposed format.

#### Exploratory analyses

The distributions of the exploratory measures mentioned in “Exploratory outcome measures” section will first be inspected, and then suitably transformed. To inspect high-dimensional data, relative log abundance/expression plots [18] and loadings/scores plots obtained from principal component analyses will be used [19]. “Remove unwanted variation” (RUV) [20] normalisation methods will be used to either remove or accommodate unwanted variation in the high-dimensional data as appropriate. Systematic patterns in the data will be investigated using clustering techniques such as the hierarchical cluster analysis coupled with inspection of heatmaps. Depending on the distribution of the exploratory outcome measure, the differences in the change in the exploratory outcome measure from baseline to endpoint between the intervention and placebo groups will be examined using either linear or generalised linear regression models. In these models, the endpoint exploratory measure will be used as the outcome and a categorical variable indicating the allocated grouping structure (intervention versus placebo) will be used as the exposure, incorporating exploratory measure at baseline and other confounding variables as covariates. Testing for multiple comparisons in high-dimensional data will be accounted for, by adjusting the *p* values obtained from these analyses using the Benjamini-Hochberg method to address the false discovery rate [5]. The exploratory findings from these analyses will inform further directions.

#### Safety analyses

Safety analyses will be based on the safety analysis set described in “Safety analysis set” section. The safety outcomes presented in Supplementary Table 2 will be summarised by group (intervention and placebo). Categorical variables will be summarised using frequencies and percentages, and the continuous variables will be summarised using appropriate central tendency and dispersion measures, and presented either as mean and standard deviation (SD) or median and 25th–75th percentile. Percentage standardized differences between the two groups will be calculated for all variables. A value ≥ 10% difference will be taken as a meaningful difference between the groups. In addition, where possible, a linear mixed model or a generalised linear mixed model will be used with the safety measure at the end of the trial as the outcome, a categorical variable indicating the group structure (intervention or placebo group) as the exposure, and the safety measure at the beginning of the trial as a covariate, also accounting for centre variability. Where appropriate, an estimate of the group effect, 95% confidence interval and *p* value will be presented. The results will be summarised in Table 5.

**Table 5:** Safety analyses results using the safety analysis set

Safety outcome measure	Values		Percentage standardized difference	Estimated difference^a^	95% Confidence Interval^a^	*P* value^a^
	Intervention (GKT137831) group (*n* = xxx)	Placebo group (*n* = xxx)				
Treatment-emergent adverse events, *n* (%)						
Abnormal laboratory analytes, *n* (%)						
Abnormal physical examination and vital signs, *n* (%)						
QT corrected electrocardiogram (ECG), mean (SD)						
Treatment-emergent qualitative ECG, *n* (%)						
Concomitant medications, *n* (%)						
Systolic blood pressure (mmHg), mean (SD)						
Diastolic blood pressure (mmHg), mean (SD)						
Heart rate (beats per minute), mean (SD)						
Weight (Kg), mean (SD)						

*SD* standard deviation

^a^ If feasible to carry out testing; in the intervention group compared to the placebo group

## Conclusion

In this article, we followed the Guidelines for the content of statistical analysis plans in clinical trials [2] to provide detailed descriptions of the statistical methods required for carrying out the statistical analysis of the GKT137831 trial. The aim of this prior statistical analysis plan is to minimise reporting bias, supporting both transparency and reproducibility of the trial findings. All statistical analyses will be conducted using R (R Foundation for Statistical Computing, Vienna, Austria; lme4 package for R for fitting linear mixed models) and Stata (StataCorp, College Station, TX, USA). Other software packages such as SPSS or SAS may be used if necessary. The results of the trial are expected to be published, and these results may be used to inform further phase 3 trials for regulatory submission.

## Supplementary information

**Additional file 1: Supplementary Table 1.** Exploratory outcome measures.

**Additional file 2: Supplementary Table 2.** Safety outcome measures.

## Data Availability

Not applicable.

## Abbreviations

eGFR: Estimated glomerular filtration rate
HbA1c: Glycated haemoglobin
HREC: Human Research Ethics Committee
ITT: Intention to treat
IWRS: Interactive Web Response System
NADPH: Nicotinamide adenine dinucleotide phosphate
PP: Per protocol
SAP: Statistical analysis plan
UACR: Urine albumin–creatinine ratio
