# Supplementary material for: Evaluating the efficacy and safety of GKT137831 in adults with type 1 diabetes and persistently elevated urinary albumin excretion: a statistical analysis plan
Source: Trials. 2020 Jun 3;21:459. doi: 10.1186/s13063-020-04404-0 (PMC7268311; doi:10.1186/s13063-020-04404-0)
Supplement: Supplementary file 1 — Additional file 1: Supplementary Table 1. Exploratory outcome measures. [file 13063_2020_4404_MOESM1_ESM.docx]

Supplementary Table 1: exploratory outcome measures

| Measure | Description |
| --- | --- |
| Epigenetic markers | DNA methylation |
| Transcriptome | total whole blood mRNA |
| Metabolomic profile |  |
| Lipidomic profile |  |
| Cystatin C |  |
| Urinary markers | Kidney Injury Molecule 1 (KIM1) and Neutrophil Gelatinase-Associated Lipocalin (NGAL) |
| Markers of inflammation | High-sensitivity C-reactive protein (hsCRP), fibrinogen, interleukin-6 (IL-6) |
