# Supplementary material for: Evaluating the efficacy and safety of GKT137831 in adults with type 1 diabetes and persistently elevated urinary albumin excretion: a statistical analysis plan
Source: Trials. 2020 Jun 3;21:459. doi: 10.1186/s13063-020-04404-0 (PMC7268311; doi:10.1186/s13063-020-04404-0)
Supplement: Supplementary file 2 — Additional file 2: Supplementary Table 2. Safety outcome measures. [file 13063_2020_4404_MOESM2_ESM.docx]

Supplementary Table 2: safety outcome measures

| Measure | Section number defined in the detailed Protocol | Abnormal ranges |
| --- | --- | --- |
| Treatment emergent adverse events | 10.4.4.2 |  |
| Abnormal laboratory analytes | 10.4.4.3 |  |
| Abnormal physical examination and vital signs | 10.4.4.4 |  |
| QT corrected electrocardiogram (ECG) | 10.4.4.5 |  |
| Treatment emergent qualitative ECG | 10.4.4.5 |  |
| Concomitant medications | 10.4.4.6 |  |
| Systolic blood pressure (mmHg) | 7.1.1 |  |
| Diastolic blood pressure (mmHg) | 7.1.1 |  |
| Heart rate (beats per minute) | 7.1.1 |  |
| Weight (Kg) | 7.1.1 |  |
